# Regulatory Implications and Control Measures for Lumpy Skin Disease, Highly Pathogenic Avian Influenza, and Foot-and-Mouth Disease in European Livestock

**DOI:** 10.3390/v18070777

**Published:** 2026-07-15

**Authors:** Carolina Baptista, Bart de Leeuw, Valentina Busin, Jobke Van Hout-van Dijk, Max Bastian, Rachael Tarlinton, Nancy De Briyne, Wiebke Jansen

**Affiliations:** 1 CIBIO (Centro de Investigação em Biodiversidade e Recursos Genéticos), InBIO (Rede de Investigação em Biodiversidade e Biologia Evolutiva), Laboratório Associado, Universidade do Porto, 4485-661 Vairão, Portugal; carolina.baptista@cibio.up.pt; 2 BIOPOLIS Program in Genomics, Biodiversity and Land Planning, CIBIO, 4485-661 Vairão, Portugal; 3 EVERI (European Veterinarians in Education, Research and Industry), 1030 Brussels, Belgium; bartdeleeuw52@gmail.com; 4 Animal Husbandry and Production, University College Dublin, D04 V1W8 Dublin, Ireland; valentina.busin@ucd.ie; 5 Pig Health Department, Royal GD, 7418 EZ Deventer, The Netherlands; j.v.hout@gddiergezondheid.nl; 6 Friedrich-Loeffler-Institut, German Federal Research Institute for Animal Health, 17493 Greifswald-Insel Riems, Germany; max.bastian@fli.de; 7 School of Veterinary, University of Nottingham, Sutton Bonington Campus, Leicestershire LE12 5RD, UK; rachael.tarlinton@nottingham.ac.uk; 8 Federation of Veterinarians of Europe (FVE), 1030 Brussels, Belgium; nancy@fve.org

**Keywords:** vaccination, immunisation, stamping out, culling, economic costs, viral diseases, livestock

## Abstract

(1) Background: Transboundary notifiable infectious viral diseases, such as lumpy skin disease (LSD), highly pathogenic avian influenza (HPAI), and foot-and-mouth disease (FMD), continue to severely disrupt Europe’s animal health and welfare, food security, and the multi-burdened livestock sector. (2) Methods: Through an extensive literature search, with data collected from the scientific literature, outbreak notifications, and European Union (EU) policy reports, this study synthesises evidence on the economic and societal impacts of preventive mass vaccination compared with stamping out for listed viral animal diseases in Europe. (3) Results: Evidence from LSD, HPAI, and FMD indicates that preventive vaccination reduces outbreak size, duration, and associated economic losses, particularly in high-risk and endemic settings. For LSD, vaccination is essential in eradication, as culling alone fails. For HPAI, evolving epidemiology supports vaccination-to-live strategies. In contrast, for FMD, despite epidemiological benefits of vaccination, the maintenance of FMD-free status without vaccination remains the dominant policy objective, constraining adoption of vaccination-to-live due to trade implications. (4) Conclusions: Overall, findings support the following recommendations: shifting toward preventive vaccination tailored by country and disease, prioritising vaccine-to-live strategies within a harmonised regulatory framework, and strengthening differentiating infected from vaccinated animals (DIVA)-based surveillance and trade-compatible frameworks, all sensible approaches to protect animal welfare and economic stability in Europe’s livestock sector.

## 1. Introduction

Transboundary notifiable infectious viral diseases, including lumpy skin disease (LSD), highly pathogenic avian influenza (HPAI), and foot-and-mouth disease (FMD), are recognised as persistent threats to animal health and welfare, food security, and livestock economic resilience in Europe and beyond [1,2,3]. Introduction and spread of these (re)-emerging infectious diseases (EIDs) is driven by a spectrum of ecological and structural factors. These include but are not limited to the following: the existence of wildlife reservoirs and migratory flyways, allowing intercontinental spread across dense production systems, facilitating farm-to-farm transmission; climate change and its consequences, such as climate-sensitivity of vectors; and increased complexity of trade networks, which amplify transboundary movement risks [2,4,5,6].

The European response to LSD, HPAI, and FMD outbreaks has primarily relied on stamping out, defined by mass culling and safe disposal, together with on-farm internal and external biosecurity measures, including movement restrictions, regionalization and compartmentalization, and increased surveillance [7,8,9]. However, it remains to be elucidated which approach balances best effectiveness at reducing disease transmission, while safeguarding animal health and welfare, and minimising the economic, ethical, and societal impacts [10,11].

**Figure 1:**
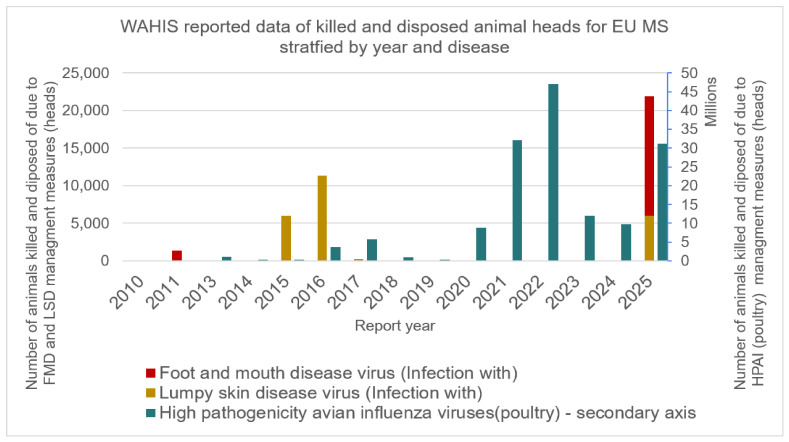
Annual number of animals culled or disposed of due to disease management measures in the EU (2010–2025), stratified by causative pathogen. The stacked bar chart displays reported animals killed and disposed of associated with highly pathogenic avian influenza (commercial poultry), lumpy skin disease, and foot-and-mouth disease, based on official WAHIS reports from the following EU Member States (in alphabetical order) to the World Animal Health Information System (WAHIS): Austria (AT), Belgium (BE), Bulgaria (BG), Croatia (HR), Cyprus (CY), Czechia (CZ), Denmark (DK), Estonia (EE), Finland (FI), France (FR), Germany (DE), Greece (EL), Hungary (HU), Ireland (IE), Italy (IT), Latvia (LV), Lithuania (LT), Luxembourg (LU), Malta (MT), Netherlands (NL), Poland (PL), Portugal (PT), Romania (RO), Slovakia (SK), Slovenia (SI), Spain (ES), Sweden (SE). Data retrieved from WAHIS (accessed on 24 February 2026).

Recently, the frequency and severity of outbreaks of HPAI and spillover to mammals, as well as the LSD and FMD, have intensified in the European Union (EU) Member States (MS). Disease management measures have led to a steady rise in animals killed and disposed of, with considerable temporal variability in disease-driven livestock losses. This pattern underscores the disproportionate impact of HPAI relative to other notifiable transboundary diseases (Figure 1). This trend emphasises the limits of reactive control models and strengthens the case for a prevention-first strategy based on a One Health framework [12]. However, animal counts do not capture differences in production output, and data on protein losses associated with disease are, to our knowledge, not systematically available.

Policymakers have increasingly pointed out the limitations of reliance on culling control strategies, particularly when vaccination is feasible and scientifically supported [13]. EU-funded collaborations have supported advances in emergency (reactive measure) and preventive (proactive strategy), including the development of differentiating infected from vaccinated animals (DIVA) vaccines and improvements at diagnostic levels. These enabled vaccination-to-live policies, allowing to rethink and reshape outbreak management [7,11].

**Figure 2:**
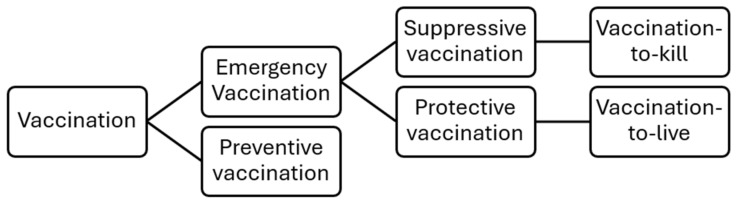
Key principles for the classification of animal vaccination strategies, adapted from WOAH Terrestrial Animal Health Code, Chapter 4.18: Vaccination (WOAH (2022), Terrestrial Animal Health Code. Available at https://www.woah.org/fileadmin/Home/eng/Health_standards/tahc/current/chapitre_vaccination.pdf (accessed on 2 July 2026)).

Consequently, vaccination, integrating not only reactive suppressive emergency vaccination, but also integrating emergency protective vaccination and proactive preventive strategies, alongside robust biosecurity and surveillance, should be considered a key option for Europe’s strategy on mitigating and responding to emerging infectious diseases (Figure 2) [13]. This shift aligns with the EU’s Preparedness Union Strategy, which promoted a proactive approach that focuses on anticipating crises rather than simply reacting to them [14].

The present review aims to explore, via a literature-based search, whether, in a context of persistent threat of listed viral animal disease outbreaks, the implementation of vaccination-to-live strategies, rather than solely as a control tool, can reduce the overall disease burden when compared with an approach relying mainly on mass culling and/or biosecurity for selected notifiable viral livestock diseases.

## 2. Materials and Methods

A structured literature search was conducted to identify evidence on the economic, societal, and animal-health impacts of vaccination and stamping-out strategies for listed viral livestock diseases in Europe, specifically, LSD, HPAI, and FMD. The literature sources included scientific articles on epidemiology, economics, welfare, and social impacts; outbreak reports and official notifications; and EU policy and regulatory documents.

For the scientific articles, predefined search strings were applied to PubMed and Scopus. These search strings combined disease-specific terms (e.g., “lumpy skin disease”, “highly pathogenic avian influenza”, and “foot-and-mouth disease”) with intervention keywords (“vaccination”, “stamping out”, and “culling”) and outcome descriptors (“economic impact”, “cost-effectiveness”, “animal welfare”, “cost-utility”, and “cost-benefit”). All terms were searched for in both Title and Abstract. Searches were restricted to English and to 2005–2026 (from 1 January 2005 to 31 December 2025). The full search strings used are provided in the Supplementary Materials.

For the context of this review, studies were included if they addressed at least one of the diseases (LSD, HPAI, or FMD), assessed vaccination and/or stamping-out strategies, and/or reported economic outcomes or implications, geographically focusing on the EU/European Economic Area (EEA).

Outbreak reports and official notifications data were retrieved from the World Animal Health Information System (WAHIS), the global animal health database of the World Organisation for Animal Health (WOAH). Policy and regulatory documents were sourced from EUR-Lex^2^. For each disease evaluated, the information extracted focused on the epidemiological outcomes (e.g., outbreak size, duration, date of occurrence), measures of prevention or control (e.g., vaccination, stamping out), and economic indicators (e.g., cost per outbreak).

Costs were defined and explicitly categorised in direct monetary costs: ((i) vaccines, logistics, administration, (ii) morbidity, mortality, culling, disposal, compensation, (iii) surveillance and diagnostics); indirect monetary costs ((i) trade bans and export losses, (ii) production losses (yield, growth), (iii) supply-chain disruption, (iv) loss of genetic capital); and non-monetary costs ((i) negative animal welfare consequences at farm and during culling, (ii) farmer and veterinary mental health, public trust and social licence, environmental impacts (carcass effluences, intermediate disposal)). Only a subset of these costs was consistently quantified in the literature, limiting direct comparison.

## 3. Results

Scientific articles and other relevant documents were selected based on their alignment with the aim of the review, particularly their contribution to better understand outbreak magnitude, vaccination strategies, stamping-out policies, and the broader policy, societal and economic implications.

Through the initial screening, *n* = 527 scientific papers were identified. After removing duplicates, *n* = 409 papers were screened, of which *n* = 9 met the inclusion criteria and were retained as core studies for the final review. All included studies were conducted within the EU/EEA, in line with the scope of this revision. Papers were excluded if the examined aspects of the selected transboundary notifiable diseases were unrelated to vaccination strategies, stamping-out, or economic and animal welfare considerations, or if they relied exclusively on data from non-EU/EEA settings. In addition to the database search, additional references were identified through citation tracking and experts’ input. Grey literature, including recent reports and assessments from organisations, such as the European Food Safety Authority (EFSA) or European Commission (EC), was also incorporated to ensure inclusion of up-to-date findings and current evidence available. These additional sources were integrated across both the core screening and as a supplementary capacity of the review, to complement and contextualise the findings.

A disease-specific synthesis followed, where these insights were reviewed and contextualised for each condition individually.

### 3.1. Lumpy Skin Disease

Lumpy skin disease virus, a member of the Capripoxvirus genus, affects and infects livestock across continents. Impacts are related to disease morbidity, which can vary from 5 to 45% towards the maximum of 100%, in a severe epidemic, resulting in substantial production losses [15].

This poxviral infection was first detected in Europe in 2015 in Greece, affecting a total of 117 farms. The disease spread further, with Bulgaria experiencing nearly 200 affected farms and the virus rapidly expanding through the Balkan region, with nearly 7000 outbreaks in 2016. Initial control measures during these outbreaks deeply relied on stamping-out policies: Bulgaria implemented total culling, whereas other countries adopted partial stamping-out or solely preventive vaccination strategies, e.g., Albania managed to successfully eradicate LSD through coordinated mass preventive vaccination campaigns by 2017. However, continued vector expansion and the influence of climate change have likely contributed to the re-emergence of this disease in 2025, with outbreaks reported in France, Italy and Spain [12,16,17,18].

Outbreaks generate significant economic burdens, affecting both farm productivity and the national livestock trade. During the outbreaks between 2016 and 2017 in the Balkan region, costs were almost 21 million euros, which included the costs for vaccination, aerial fumigation and compensation to the farmers, this last one being the highest cost [16]. The 2025 outbreaks in France reached a cost of 2.4 million euros following compensation of 42 farmers [17].

Culling policies seem to be outdated once we investigate both modelling and field studies. EFSA’s kernel-based modelling for Greece and Bulgaria demonstrated that 95% of farm coverage by vaccination could aim for nearly 100% outbreak extinction when vaccination was used for prevention. Similarly, field sampling from Greece estimates an approximate 80% effectiveness of vaccination [19,20]. The Balkan outbreak eradication demonstrated that annual and high-coverage mass vaccination was decisive, with field estimates of 70% of vaccine effectiveness, and highlighted that no country has eliminated LSD without vaccination [17,21]. In line with this evidence, the latest EFSA assessment on vector-borne diseases, including LSD, concludes that culling, i.e., stamping-out, is less effective than vaccination when used alone, and cannot control outbreaks as a stand-alone measure. Instead, it only plays a supportive role when combined with vaccination and strict movement restrictions [22].

The findings summarised in Table 1 highlight the comparative strengths and uncertainties of different control measures for LSD. Preventive vaccination emerges as the most effective strategy, but results in the loss of LSD-free status. However, together with what was previously mentioned, an integrated approach, combining vaccination, surveillance, biosecurity, and movement controls, seems to provide a more sustainable outcome, aiming at disease control in balance with economic costs, trade implications, and animal welfare considerations (Table 1) [16,19,21,22].

Currently, LSD is categorised as an A+D+E disease under the Animal Health Law (Regulation (EU) 2016/429). Confirmation of LSD triggers a series of mandatory control measures, including the stamping out of infected herds, the establishment of 20 km protection zones and 50 km surveillance zones, movement bans, strict biosecurity, and enhanced surveillance. In addition, emergency protective vaccination may be deployed under Regulation (EU) 2023/361 [7] to curb the spread of disease. These actions, under the EU Animal Health Law, are designed to prevent LSD transmission and safeguard livestock trade [18,22].

For future outbreaks, strategies should prioritise DIVA-compatible vaccines, genomic surveillance, and sustained regional coordination to minimise reintroductions, especially where vector pressure and climate trends favour spread [12,19].

Data also indicated that mass vaccination halted the spread in South-Eastern Europe and was more favourable than stamping out in at-risk, vector-driven settings. Following EFSA’s scientific advice published in August 2016, vaccination was recommended to minimise the number of LSD outbreaks in regions already affected or at risk. Evidence demonstrates that vaccination-to-live is necessary, as no country has been able to eliminate LSD without vaccination [20,23].

### 3.2. Highly Pathogenic Avian Influenza

For three decades, Europe’s poultry sector has been disrupted repeatedly by outbreaks of HPAI, an Influenza A virus. At a policy level, HPAI control was first harmonised in Europe in 1992. However, practical implementation of control measures only followed the outbreak in Italy in 1999, after several incursions of both HPAI and low-pathogenic avian influenza (LPAI) circulation in Europe. These measures were later updated in 2005, introducing vaccination as a potential preventive tool and not only as an emergency response [24,25].

Since the implementation of WAHIS in 2005, tens of thousands of HPAI events have been reported across Europe, with marked intensification in recent years. In the last quarter of 2025 alone, Europe notified 2896 HPAI detections (368 poultry, 74 captive birds, 2454 wild birds), affecting a total of 11 million poultry in the space of a few months through mortality or depopulation [26]. Similar patterns have been occurring since the late 1990s, underlining the need to re-evaluate disease control methods and raising concerns regarding animal welfare and ethical implications [27,28]. Furthermore, HPAI has gained increasing relevance within the One Health context due to its association with spill-over to mammals, including occasional human infections, thereby reinforcing its zoonotic risk and broader public health relevance [29].

Currently, HPAI is categorised as an A+D+E disease under the Animal Health Law (Regulation (EU) 2016/429). The default control strategy is eradication, while vaccination is seen mainly as an emergency tool and is strictly regulated by the Animal Health Law, with trade restrictions imposed in infected and surrounding areas [30,31]. Recent EU/EEA strategies have included strict biosecurity, targeted sectoral preventive vaccination, and enhanced surveillance, along with wildlife management and occupational protection [26]. To mitigate trade disruption, control strategies such as zoning, regionalisation and compartmentalisation were also applied [30].

Italy played a shifting role in shaping Europe’s approach to HPAI control in the late 1990s. Capua and Marangon documented in 2006 the implementation of emergency vaccination during the 1999 outbreak, combined with restriction measures in the affected areas. This strategy, supported by WOAH, demonstrated that vaccination can be applied without jeopardising international trade, challenging the prevailing reliance on stamping-out policies [27,32]. However, silently infected animals and economic concerns about trade restrictions continue to be the most important arguments against HPAI vaccination globally [33,34].

The economic impact of HPAI outbreaks at the EU/EEA level has been critical. In 1999, Italy’s total losses were estimated at 507 million euros, divided into 112 million euros in direct costs and 395 million euros in consequential losses due to, e.g., trade implications, disposal, etc., a result of the absence of preventive measures at the time [27,28]. Poland’s 2021 HPAI outbreak incurred the culling of more than 10 million birds, with an incurred annual cost of 250 million euros, including indirect losses [35]. In 2024 alone, HPAI control measures (from implementation to compensation) in Austria cost an estimated 3 million euros, while modelling studies suggested that a preventive vaccination strategy targeting “very high-risk” areas would cost up to 2.4 million euros and could be a more cost-effective measure, if losses were to be avoided [9]. Despite the economic and animal welfare impacts mentioned, control of HPAI in Europe has continued to rely on stamping-out measures, resulting in mass depopulations, e.g., 16 million birds in Italy (1999), 30 million in the Netherlands (2003), and more than 10 million in Poland during a single outbreak in 2023, and hundreds of millions culled across Europe between 2020 and 2023 [26,27,35,36]. The limitations of late depopulation strategies were highlighted by Fusaro et al. [37], who reported that a 14-day delay in mass culling prompted the spread of H7N7 in Italy in 2013.

Although vaccination has traditionally been used for only high-value flocks or in emergency situations, a growing body of evidence consistently supports its use as a preventive tool alongside other biosecurity measures due to the changing epidemiology (Table 2), whereas in the past, the virus circulated in poultry, the main reservoir of H5-viruses nowadays is wild and migratory birds, severely limiting the possibility of reasonably eradicating the reservoir [38]. Preventive strategies, such as reducing animal density and targeted vaccination, have shown effectiveness in southwest France, where a reduction in animal density was associated with a decrease in outbreaks from 331 outbreaks in 2021–2022 to 20 outbreaks in 2022–2023 [39]. In addition, large-scale duck vaccination was implemented in France in October 2023, and HPAI outbreaks in poultry during the 2023–2024 season were reduced to only 10. The programme, which had estimated costs of over 100 million euros, was the first mass-vaccination initiative of its kind in the European Union and required the vaccination of roughly 64 million ducks [40]. Modelling studies further suggest that, in the absence of vaccination, the number of outbreaks would have been substantially higher [41]. Importantly, these epidemiological gains are likely economic benefits, as compensation costs, exceeding 1.6 million in France, could potentially be reduced [42].

These findings align with broader evidence showing that stamping out, biosecurity, or vaccination alone face limitations. Overall, an integrated approach of the three appears to provide a more balanced and sustainable strategy, improving HPAI control, while reducing economic and societal burdens (Table 2) [7,9,43,44,45,46]. Vaccination costs are relatively low (0.34 € per dose and up to 0.26 € for administration per animal), while compensation for culled birds reaches up to 10.63 € per animal (Table 2). These figures highlight the economic trade-off between preventive and reactive strategies. Advances in vaccine development, such as in ovo and vector-based vaccines, have the potential to further reduce costs and constraints by enabling early and large-scale implementation, strengthening vaccination as a cost-effective component of integrated control approaches (Table 2) [9,43].

While stamping out at infected establishments remains legally compulsory, even when vaccination is applied, vaccination limits spread and downstream losses, and must be paired with reinforced surveillance and movement conditions set by Delegated Regulation (EU) 2023/361 [7]. When additional costs, such as compensation or carcass disposal, are considered, a combined approach (preventive vaccination, biosecurity and surveillance) may be more cost-effective than repeated stamping-out in densely populated poultry regions with recurrent incursions. Evidence supports progressive adoption of vaccination-to-live strategies, particularly in dense poultry production systems with recurrent outbreaks.

### 3.3. Foot-And-Mouth Disease

Foot-and-mouth disease, caused by a highly infectious picornavirus, is one of the most consequential transboundary livestock diseases, with implications extending from animal health to the global economy. FMD-free without vaccination status has been key for market access and is considered the primary objective of livestock health, enabling highly lucrative, strict global trade markets for meat and dairy. However, it requires a massive, coordinated effort in biosecurity and surveillance, and its vulnerability increases during outbreaks, as even a single outbreak can result in a suspension of status [47]. When incursions occur, this status is suspended, and trade bans produce such severe economic losses that often exceed direct production impacts [48]. The 2001 UK outbreak illustrates this economic damage, which led to 8 billion British pounds (~12.8 billion euros) in losses, mainly as a consequence of trade bans and compensation for the culling of approximately 6 million animals [49]. Similarly, even limited outbreaks, such as the ones modelled for Denmark, triggered disproportionate trade disruptions reaching hundreds of millions of euros [8].

Stamping out remains the default control strategy for FMD worldwide, which entails mass depopulation and raises societal and ethical concerns. For example, in the UK, modelling indicates that solely relying on stamping out resulted in more infected premises (145) and a longer duration of the epidemic (111 days), whereas introducing early emergency vaccination, from day 10, reduced the infected premises to 80 and shortened the outbreak by 30 days [50]. Consistently, Bradbury et al. demonstrated that all vaccination strategies outperformed relying on culling strategies alone. These would translate to an expected decrease in outbreak duration, fewer livestock culled and millions of pounds saved, highlighting the importance of contingency planning [51].

In 2025, Wagner suggested, through a modelling study focusing on FMD outbreak control strategies and their economic efficiency in Denmark, that stamping-out remained cost-effective for small outbreaks, whereas vaccinate-to-kill strategies were preferable in large-scale epidemics, as they can be less costly overall (Figure 2) [8]. Cabezas et al. 2022, in a retrospective analysis of factors influencing the time to recovery WOAH FMD-free status, showed that stamping out or vaccination-to-kill strategies regained FMD-free status without vaccination status in around 6 months, when compared to 21 months if vaccination-to-live and retention strategies were applied [48].

Currently, FMD is categorised as an A+D+E disease under the Animal Health Law (Regulation (EU) 2016/429). Despite growing concerns, stamping out is still the main strategy of control of FMD, as observed in Germany, Hungary and Slovakia in 2025. In accordance with Regulation (EU) 2016/429 [30] and Delegated Regulation (EU) 2020/687 [31], these countries proceeded to the culling of the remaining animals in the affected establishments and implemented temporary restriction and surveillance areas. FMD-free without vaccination status was re-established for Germany three months after the detection of disease, due to the limited scale, location, and spread, which never led to emergency vaccination, neither suppressive nor protective (Figure 2). In contrast, both Hungary and Slovakia took six months to regain FMD-free status without vaccination and had to apply emergency suppressive vaccination to limit the spread and reduce clinical symptoms on infected farms. In early 2026, Cyprus and Greece reported FMD outbreaks. While Cyprus opted for emergency protective vaccination, Greece implemented control measures, including restricted zones on the island Lesvos, and additional measures, including restrictions on movements of susceptible animals from the further restricted zone [52].

Preparedness and resource allocation, in terms of robust veterinary capacity, were considered to be key for a quick restoration of FMD-free status without vaccination, as is the case for higher-income countries (gross national income (GNI) from The World Bank income classification system), which recover up to six times faster [49]. Future strategies should emphasise preventive measures, such as securing vaccination capacity and development of next-generation vaccines, to overcome current limitations, e.g., to provide longer duration of immunity, to allow safer production, and/or to enhance DIVA capabilities, and prevent the risk of large-scale outbreaks leading to the genuine impossibility to regain the FMD-free status without vaccination [49].

The findings summarised in Table 3 highlight the distinct strengths and limitations of FMD control measures. Stamping out ensures rapid containment, involving high economic and ethical costs, particularly in large outbreaks. Vaccination strategies and policies, such as vaccine-to-kill or vaccine-to-live, have shown the capacity to reduce the outbreak size and duration. Nevertheless, vaccination introduces trade-offs, including delays in regaining WOAH FMD-free without vaccination status and increasing logistical challenges. Overall, evidence supports a combined approach that balances epidemiological effectiveness with economic and societal considerations (Table 3) [53,54]. While advances in vaccine technologies (e.g., enhanced inactivated vaccines, viral vectored platforms, and empty capsid particles) may strengthen vaccination as a scalable component of FMD control, logistical constraints remain. For example, maintaining an adequate vaccine stock and managing restocking delays of around 21 days after disinfection, which may be shorter in some scenarios, emphasised the logistical challenges of outbreak recovery and the need for preparedness in Scotland [54,55,56,57]. Resource availability and timing directly affect control efficiency. Overall, planning vaccine stocks and recovery timelines is critical for effective FMD management [48,49,50], as well as the availability of commercial FMD diagnostic kits based on DIVA [58].

The literature showed that early emergency vaccination with adequate vaccine stocks improved both epidemiological and economic outcomes compared with stamping out alone, but the WOAH FMD-free status recovery without vaccination considerations matters. To achieve this status, a country must prove no infection has occurred for the past 12 months, prohibit vaccination, provide evidence of surveillance, and manage animal identification/traceability. Article 8.8.11 of WOAH’s terrestrial code allows as early as three months after the last vaccination without necessitating the killing of all vaccinated animals. Vaccination-to-kill often regains status faster than vaccination-to-live, which harbours inherent challenges for animals with a longer lifespan, such as cattle. Whether the slaughter of otherwise healthy animals just to regain the WOAH status would be legally admissible remains to be elucidated [59]. Each EU country must decide based on the specific situation and a detailed analysis (e.g., risks and cost–benefit ratio) if emergency vaccination is appropriate for their situation. Vaccination-to-live remains constrained and is unlikely to be prioritised under current trade frameworks despite epidemiological advantages.

## 4. Discussion

Aligning with the current regulation established by the Animal Health Law, this literature research indicated that stamping out was used as the default strategy for the control of LSD, HPAI and FMD in recent decades at the EU/EEA level, with vaccination evaluated as an emergency occurrence or as a preventive tool in high-risk areas [31]. A limitation of this review is that information on non-monetary costs was not systematically identified in the literature reviewed. Nevertheless, these aspects remain relevant considerations when evaluating disease-control strategies and their negative consequences on animal welfare, mental health, and the environment.

However, the majority of the studies also highlighted that vaccination remains a central component for effectiveness in terms of disease management and clinical perspective, but also for ethical and economic reasons [50,51]. The analysed implementation of vaccination-to-live policies differs substantially between diseases and is influenced by pathogen ecology, epidemiological characteristics, and trade frameworks. LSD, field experience from South-Eastern Europe demonstrated that high-coverage vaccination campaigns were essential for disease control and eventual eradication, while EFSA assessments concluded that stamping-out alone is insufficient to control outbreaks of this vector-borne disease [16,19,20,21,22,23]. For HPAI, increasing evidence indicates that the persistence of virus circulation in wild bird populations has fundamentally changed the epidemiological situation, resulting in recurrent incursions into poultry despite extensive eradication efforts and supporting consideration of vaccination-to-live approaches as part of integrated control programmes [26,34,38,39]. In contrast, although emergency suppressive vaccination can improve epidemiological and economic outcomes during FMD outbreaks, policy decisions continue to be strongly influenced by the objective of maintaining or rapidly regaining WOAH FMD-free without vaccination status because of its importance for international trade, limiting the wider adoption of vaccination-to-live strategies [8,41,48,59].

Preventing disease burden was consistently shown to be more efficient than only responding reactively to detected outbreaks, reducing animal suffering, limiting pathogen spread, and safeguarding production systems [10,11]. Suggestions for future control measures should prioritise vaccination planning and investment in vaccine development, cost-optimised strategies, and genomic monitoring, to detect evolving genotypes and adaptation, to anticipate spillover events and prevent market disruptions [35].

The recent re-categorisation of bluetongue virus (BTV) disease to Category D+E disease has been considered an evidence-based adjustment towards realistic and sustainable control tools aligned with current epidemiological trends, accepting the impossibility of controlling vector movements and abandoning BTV eradication. The strategic value of vaccination against BTV has been further emphasised as vaccination has repeatedly shown to have positive benefit–cost ratios and to reduce outbreak magnitude and duration [60,61,62,63]. With focus on surveillance, biosecurity, and mass vaccination, the re-categorization has created conditions for rapid detection systems (e.g., Sweden’s bulk-milk monitoring) to be more effectively and sustainably integrated into future mitigation and control strategies [64].

Despite the advantages stated above, concerns remain regarding vaccine effectiveness, trade implications, and, consequently, associated financial and logistical constraints, while regulatory uncertainty may also disincentivise the development of innovative vaccines [9,13,48]. Transparency is then required, through clear communication, particularly regarding the different vaccine technologies available, the different administration routes, and how these can lead to differences in protection, immune response, and duration of immunity [49]. Hence, these show the relevance of DIVA-compatible tools to be able to distinguish between infected and vaccinated animals [11]. WOAH’s free status without vaccination does not fully consider the capabilities of today’s DIVA vaccines and the associated diagnostic kits, allowing demonstration of freedom from virus circulation regardless of vaccination status.

Advances in vaccine development may further facilitate large-scale implementation and improve the cost-effectiveness of the vaccination programmes, reinforcing this method as a sustainable alternative to repeated culling measures.

Biosecurity and sanitation measures, e.g., controlling animal movements and maintaining environmental hygiene, have been shown to reduce the risk of disease introduction and transmission within the livestock populations. The introduction of these measures has been considered to be key in safeguarding animal health and welfare [65].

While vaccination is being considered as a preventive tool and as a preferred approach, it needs other measures implemented simultaneously, e.g., biosecurity measures. Stamping out rapidly suppresses infection during early outbreak stages. However, its repeated use carries substantial economic, ethical, logistical, and psychological costs, including compensation expenses, carcass disposal, supply chain disruption, animal welfare concerns, and long-term stress among farmers and veterinarians. Therefore, integrated approaches combining vaccination with targeted culling may offer the most balanced strategy when adapted to the epidemiological context and key factors, e.g., pathogen characteristics, as illustrated by the response to the LSD outbreak in France in 2025 [8,18,37,39,48].

European and international policy discussions, supported by scientific opinions, mandates, and professional consensus statements, underscore the socio-economic value of vaccination, the importance of DIVA-compatible tools, and the importance of establishing a harmonised and standardised regulatory pathway to ensure vaccination is not a barrier to safe trade [13].

## 5. Conclusions

As a result of the current global epidemiological changes, the introduction to and spread of transboundary viral diseases within the European Union is an ongoing risk to animal health and welfare, both from a societal and economic perspective. The limits of current economic evidence for interventions could be addressed in standardised EU-wide economic reporting templates, fostering the establishment of scenario-based modelling, including trade rules, integrating One Health externalities. To address these risks, there is still a need to improve and better implement preventive and control measures within the EU/EEA. These include supporting vaccine innovation and ensuring vaccine availability through the promotion of vaccination programmes. Equally, it is important to guarantee a supportive legal framework that reduces trade barriers and enables a vaccination-to-live strategy for emergency vaccination without compromising official freedom from disease status. France’s experience with duck vaccination demonstrates the feasibility of this approach, as trade disruptions were temporary and solved while vaccination programmes continued. Disease re-categorisation and granularity of status to reflect current realities may be worth consideration. Protecting animal welfare and promoting economic stability across the livestock sectors requires enhancing Europe’s preparedness towards transboundary infectious diseases through a One Health lens.

## Figures and Tables

**Table 1:** Comparison of control strategies (stamping-out and vaccination) for LSD in Europe. The table compares stamping-out, preventive vaccination and integrated approaches, highlighting differences in assumptions, costs, and implementation impacts.

Strategy	Limitations	Direct Costs Impacts (Quantitative)	Non-Monetary Impacts (Qualitative)	Remarks	Key Reference(s)
Stamping-out (1)	Reporting bias			Culls without halting vector-borne spread Prolonged restrictions	[19,21]
Preventive vaccination (2)	Loss of LSD-free status	Albania in 2017, with direct costs of 5.3 € million	Welfare Strong field effectiveness (~80%) Broad acceptance	Dominant when compared to stamping out in at-risk regions	[16,21]
Integrated approaches (1 and 2, including biosecurity)	Outcomes depend on vaccine logistics and vector/biosecurity uncertainties	Bulgaria in 2017 with direct costs—8.6 € million	Welfare Strong public confidence Minimal trade disruption	Preferred sustainable package in risk areas	[16,21]

**Table 2:** Comparison of control strategies (stamping-out, biosecurity, and vaccination) for HPAI in Europe. The table compares stamping out, biosecurity, and vaccination, highlighting differences in assumptions, costs, and implementation impacts.

Strategy	Limitations	Direct Costs Impacts (Quantitative)	Non-Monetary Impacts (Qualitative)	Remarks	Key Reference(s)
Stamping-out (1)	Limited impact in high-density areas Delays from limited culling/disposal capacity and late detection Big species effect (ducks/turkeys being highly infectious) Models exclude backyards and simplify reality	Compensation per species: €0.98–€10.63/bird (broiler chickens—0.98 €/bird, ducks—2.09 €/bird, turkey—10.63 €/bird, inside layers—1.98 €/bird, outside layers—2.14 €/bird, ready-to-lay layers—2.00 €/bird, ready-to-lay breeders—5.84 €/bird, breeders (parents stock)—7.00 €/bird) Cleaning and Disinfection: 1.90 €/bird Transport: 0.66 €/bird Tracing: 501 €/farm Screening: 541 €/farm	High culling Disposal burden Staff exposure Public concern	Costly in recurrent waves May control small, contained events	[43,44]
Biosecurity (2)	Variability within farms Outdoor flocks Wetlands proximity Dense areas High movement networks increase risk		Welfare Environment Risk of failure in high-risk seasons	Rarely sufficient alone in high-risk areas	[45]
Preventive vaccination (3)	Vaccination does not compensate for weak biosecurity (silent circulation) Effectiveness depends on species, coverage, immunity, field/lab gap	Total cost per animal—1.52–4.06 € depending on scenario Vaccine cost per dose: 0.34 € Application: 0.04–0.2 €6/bird Post-vaccination surveillance/monitoring is 152.5 €0/farm, >50% of total cost in broad free-range programmes	Welfare Surveillance obligations Movement conditions apply	Likely cost-effective vs. repeated culling in dense areas	[9,44]
Integrated approaches (1, 2, and 3)	Control is an integrated matter Model outcomes assume baseline biosecurity		Best overall welfare Stronger public acceptance Robust surveillance required	Preferred in dense poultry belts with recurrent waves	[9,43,45]

**Table 3:** Comparison of modelled control strategy costs (stamping-out and vaccination-to-live) for FMD in Europe. The table compares stamping-out and vaccination, highlighting differences in assumptions, costs, and implementation impacts.

Strategy	Limitations	Direct Costs (Quantitative)	Non-Monetary Impacts (Qualitative)	Remarks	Key Reference(s)
Stamping-out (1)	Large-scale culling, causing ethical and social issues Assumes movement restriction compliance, which is unrealistic Delays in detection worsen outcomes	862 £ million median (range 169–1701 £ million), which translate approximately to 750 € million median (range 147–1480 € million) *	Large culls Trade disruption Rapid WOAH status recovery if contained	Baseline strategy Expensive if outbreak expands	[54]
Protective vaccination-to-live(2)			Welfare May delay WOAH status recovery (retain)	Economically beneficial in severe outbreaks Status considerations apply	[54]
Integrated approaches (1 and 2)	Trade restrictions double Large vaccine stocks Delays lead to larger outbreaks and more costs	417 £ million median (range 155–1455 £ million), which translate approximately to 363 € million median (range 135–1265 € million) *	Moderate culling Faster WOAH recovery than retain	Often preferable in severe scenarios if vaccine logistics are ready	[48,54]

* Costs from the 2011 outbreak in Scotland, estimated in euros based on the value of £ at the time.

## Data Availability

The original data presented in the study are openly available in the World Animal Health Information System (WAHIS) by World Organisation for Animal Health (WOAH), World Animal Health Information System (WAHIS) at https://wahis.woah.org/#/home (accessed on 15 January 2026).

## Supplementary Materials

The following supporting information can be downloaded at: https://www.mdpi.com/article/10.3390/v18070777/s1, Search strings used for this revision.

## Abbreviations

The following abbreviations are used in this manuscript:

BTV	Bluetongue Virus
DIVA	Differentiating Infected From Vaccinated Animals
EC	European Commission
EEA	European Economic Area
EFSA	European Food Safety Authority
EU	European Union
FMD	Foot-and-Mouth Disease
FOD	Freedom of Disease
GNI	Gross National Income
HPAI	Highly Pathogenic Avian Influenza
LSD	Lumpy Skin Disease
MS	Member States
UK	United Kingdom
WOAH	World Organisation for Animal Health
WAHIS	Animal Health Information System
