# Supplementary material for: Regulatory Implications and Control Measures for Lumpy Skin Disease, Highly Pathogenic Avian Influenza, and Foot-and-Mouth Disease in European Livestock
Source: Viruses. 2026 Jul 15;18(7):777. doi: 10.3390/v18070777 (PMC13431630; doi:10.3390/v18070777)
Supplement: Supplementary file 1 [file viruses-18-00777-s001.zip › viruses-4380033-supplementary.pdf]

Supplementary Materials:

Search strings used for this revision are as follows:

- LSD (TITLE-ABS-KEY (("lumpy skin disease" OR LSD) AND (vaccination OR immunization OR "vaccination-to-live" OR "vaccinate-to-live" OR "stamping out" OR culling) AND ("economic impact" OR economics OR "cost-benefit" OR "cost effectiveness" OR "cost-effectiveness" OR "animal welfare" OR "welfare")));
- HPAI (TITLE-ABS-KEY (("highly pathogenic avian influenza" OR HPAI) AND (vaccination OR immunization OR "vaccination-to-live" OR "vaccinate-to-live" OR "stamping out" OR culling) AND ("economic impact" OR economics OR "cost-benefit" OR "cost effectiveness" OR "cost-effectiveness" OR "animal welfare" OR "welfare")));
- FMD (TITLE-ABS-KEY (("foot-and-mouth disease" OR FMD) AND (vaccination OR immunization OR "vaccination-to-live" OR "vaccinate-to-live" OR "stamping out" OR culling) AND ("economic impact" OR economics OR "cost-benefit" OR "cost effectiveness" OR "cost-effectiveness" OR "animal welfare" OR "welfare"))).
